# Relationship between Abdominal Circumference and Incidence of Hypotension during Cesarean Section under Spinal Anesthesia

**DOI:** 10.1155/2020/6547927

**Published:** 2020-08-18

**Authors:** Pattaraleeya Thomard, Sunthiti Morakul, Nichawan Wirachpisit, Wichai Ittichaikulthol, Chawika Pisitsak

**Affiliations:** Department of Anesthesiology, Faculty of Medicine, Ramathibodi Hospital, Mahidol University, Salaya, Thailand

## Abstract

**Background:**

Enlarged uterus can compress the inferior vena cava and cause hypotension when lying supine. Previous studies have shown a positive association between the abdominal circumference and size of the uterus. Therefore, the aim of this study was to evaluate the relationship between abdominal circumference and incidence of hypotension during cesarean section under spinal anesthesia.

**Methods:**

The study cohort comprised women undergoing cesarean section under spinal anesthesia. Patients were divided into two groups according to the median abdominal circumference (<101 cm and ≥101 cm). Hypotension was defined as a systolic blood pressure of <90 mmHg or mean arterial pressure of <65 mmHg. The primary outcome of this study was the relationship between the incidence of hypotension and the abdominal circumference after spinal anesthesia in term pregnant women.

**Results:**

The study cohort comprised 100 women. The incidence of hypotension did not differ between the groups (71.42% in the smaller vs. 78.43% in the larger abdominal circumference group, *p*=0.419). However, the decrease in mean arterial pressure and its percentage decrease from baseline were greater in the larger than in the smaller abdominal circumference group (change in mean arterial pressure: 28.33 mmHg (18.66–33.67) in the smaller vs. 36.67 mmHg (23.34–43.34) in the larger abdominal circumference group, *p*=0.004; percentage decrease: 31.41% (22.74–39.22) in the smaller vs. 38.47% (28.00–44.81) in the larger abdominal circumference group, *p*=0.022).

**Conclusions:**

Large abdominal circumference in pregnancy is associated with greater decreases in mean arterial pressure from baseline. However, the incidence of hypotension defined by standard criteria did not differ between larger and smaller abdominal circumference groups.

## 1. Introduction

A high level of sensory block after spinal anesthesia increases the incidence of hypotension in pregnant women [1]. High abdominal pressure is one of the factors that affect the cephalad spreading of local anesthetic agents [2, 3]. Many of the term pregnant women developed varying grades of abdominal hypertension because of their enlarged uteri [2, 4]. However, it is impractical to measure abdominal pressure and attempts to do so can increase the risk of infection. Previous studies have demonstrated associations between larger abdominal circumference (AC) and higher abdominal pressure [5] and level of sensory block [6, 7]. Therefore, it is rational to use AC as a surrogate for abdominal pressure in this study. Another explanation for hypotension after spinal anesthesia is supine hypotensive syndrome, which is when hypotension while lying supine results from the enlarged uterus directly compressing the inferior vena cava and descending aorta. Thus, the incidence of hypotension may be greater in pregnant women with larger uteri than in those with smaller uteri. We hypothesized that the increased AC, which was previously found to be associated with the increased abdominal pressure and the enlarged uterus, was related to the increased incidence of hypotension after spinal anesthesia. The aim of this study was to evaluate the relationship between the AC and incidence of hypotension during cesarean section under spinal anesthesia.

## 2. Materials and Methods

This prospective observational study was approved by the Ethical Clearance Committee on Human Rights Related to Research Involving Human Subjects, Ramathibodi Hospital (04-60-24). Written informed consent was obtained from all patients. This study was conducted at Ramathibodi Hospital, Bangkok, Thailand, from March to December 2018. The study was registered at TCTR20181203001. Inclusion criteria were term pregnant women aged between 15 and 45 years with American Society of Anesthesiologists physical status 2-3 who underwent cesarean section under spinal anesthesia. Exclusion criteria were as follows: high-risk pregnancy, for example, placenta previa, abruptio placentae, eclampsia or preeclampsia, multiple pregnancy, and cardiovascular comorbidities.

On the day of surgery, the AC of all patients was measured at the umbilical level in the supine position by one operator throughout the study. The spinal anesthesia was conducted with the standard technique by an attending anesthesiologist. In the operating room, all patients were monitored with standard monitoring, including automatic noninvasive blood pressure monitoring, three-lead electrocardiography, and pulse oximetry before initiation of spinal anesthesia. Then, spinal anesthesia was administered in the lateral position using 0.5% hyperbaric bupivacaine, doses being adjusted by the anesthesiologist performing the procedure. A 27-gauge Quincke-tip spinal needle was used. The level of spinal anesthesia was assessed by pinprick sensation. The operative table was adjusted to achieve *T*4 level of spinal anesthesia. Crystalloid fluids (500 mL) were infused with a coloading technique. Fluid management during the perioperative period was titrated by the anesthesiologist. The blood pressure including systolic blood pressure, diastolic blood pressure, mean arterial pressure, and heart rate were obtained at baseline and every minute for 10 minutes after spinal anesthesia by noninvasive technique using Nihon Kohden's Life Scope G5™. The intravenous ephedrine was titrated as needed to achieve a mean arterial pressure (MAP) of at least 65 mmHg. The dosage of ephedrine was recorded. In this study, hypotension was defined as a systolic blood pressure of less than 90 mmHg or a MAP of less than 65 mmHg. Significant hypotension was defined as a 40% decrease in MAP from baseline [8].

The primary outcome of this study was the relationship between the incidence of hypotension and the abdominal circumference after spinal anesthesia in term pregnant women. The patients were divided into smaller abdominal circumference group (smaller AC group) and larger abdominal circumference group (larger AC group) using the median value of the AC in this study.

## 3. Statistical Analysis

Nonparametric continuous variables are presented as median and interquartile range (IQR). Categorical variables are presented as number (percentage). The *χ*^2^ or Fisher's exact test was used to compare categorical variables. Quantitative variables were compared using the *t*-test when data were normally distributed and the Mann–Whitney *U* test when data were nonnormally distributed. Repeated-measure ANOVA was used to determine the changes in MAP over time between larger and smaller AC groups. Univariate analysis and multivariate regression analysis were used to identify independent risk factors for significant hypotension. Spearman's rho was used to test correlations between AC and maximal MAP change from baseline. *p* < 0.05 was considered to denote statistical significance.

All statistical analyses were performed using SPSS software, version 24, for Windows (IBM, Armonk, NY, USA).

The sample size calculation was based on the previous study that found the correlation coefficient of 0.36 between maximal dermatomal level after spinal anesthesia and AC. The minimum of the sample size to identify this relationship was 58 to achieve 80% power with a significant level of 0.05. We increased the sample size to 100 due to the assumption of the close correlation between the maximal dermatomal level and the incidence of hypotension [1].

## 4. Results

The study cohort comprised 100 pregnant women. No one was excluded from the study after the inclusion criteria had been met. The median (IQR) of abdominal circumference was 101 (95–109) cm. Finally, a total of 49 cases were in the smaller AC group and 51 cases were in the larger AC group. The patient characteristics were similar in the two groups except for age, the patients being older, and the neonatal weight greater in the larger AC group (Table 1). The anesthetic level was tested and recorded until the skin was incised. The proportion of patients who had anesthetic level other than *T*4 before skin incision did not differ between groups.

**Figure 1:**
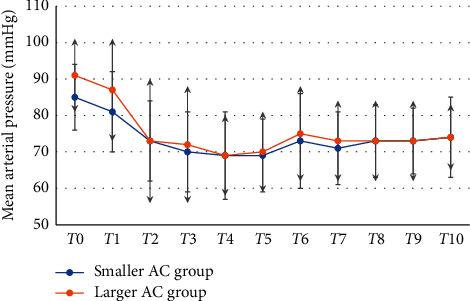
Mean arterial pressure from baseline until 10 minutes after spinal anesthesia. Data are mean ± SD of mean arterial pressure. Data were analyzed using repeated-measure ANOVA. *T*0 = baseline mean arterial pressure before spinal anesthesia and *T*1–*T*10 = time after spinal anesthesia at 1-minute interval. AC = abdominal circumference; *∗p* value < 0.05 between the smaller and larger AC groups.

**Figure 2:**
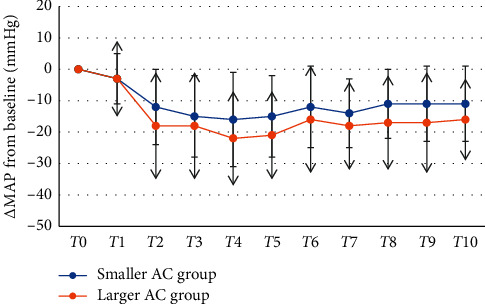
Mean arterial pressure difference from baseline until 10 minutes after spinal anesthesia. The values were calculated as MAP at *T*1, *T*2, *T*3,…, *T*10 subtracted by MAP at *T*0. Data are mean ± SD of mean arterial pressure difference. Data were analyzed using repeated-measure ANOVA. *T*0 = baseline mean arterial pressure before spinal anesthesia and *T*1–*T*10 = time after spinal anesthesia at 1-minute interval. AC = abdominal circumference; *∗p* value < 0.05 between the smaller and larger AC groups.

When analyzing the hemodynamic changes from baseline (*T*0) and over the following 10 minutes at one-minute interval after spinal anesthesia (*T*1, *T*2, *T*3, *T*4, *T*5,…, *T*10), MAP was not significantly different between the larger AC group and the smaller AC group (*p*=0.19) (see Figure 1). There was a statistically significant effect on the decrease in MAP from baseline between the larger AC group and the smaller AC group (*F* (1.98) = 4.95; *p*=0.02) (see Figure 2). Heart rate was not different between groups (*p*=0.94).

**Figure 3:**
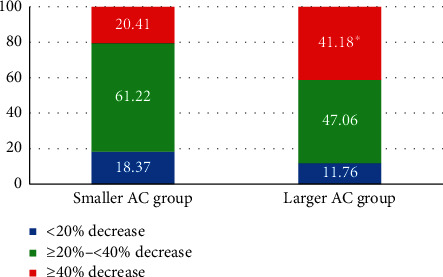
Stratification by percentage decrease in mean arterial pressure from baseline in each study group. Data are presented as percentages. AC = abdominal circumference; MAP = mean arterial pressure; *∗p* value < 0.05 between smaller and larger AC groups.

The overall incidence of hypotension did not differ between groups; however, the maximal decrease in MAP after spinal anesthesia from baseline (MAP at *T*0–lowest MAP at any time point from *T*1 to *T*10) was higher in the larger AC group (see Table 2). Although the MAP decreased more in the larger AC group, the incidence of bradycardia and tachycardia and total dosage of ephedrine were similar between the groups (see Table 2). The percentage change in MAP according to the study group is shown in Figure 3. The incidence of significant hypotension, defined as a decrease in MAP of more than 40% from baseline, was higher in the larger AC group than in the smaller AC group (20.41% in the smaller AC group vs. 41.18% in the larger AC group, *p*=0.025). Among the 31 women who developed significant hypotension, larger AC was found to be an independent risk factor for significant hypotension (odds ratio: 3.67, 95% CI: 1.01–13.28, *p*=0.04) after adjusting for covariates of age, body mass index, baseline heart rate, American Society of Anesthesiologists physical status, intraoperative fluid, blood loss, and dose of hyperbaric bupivacaine using multiple logistic regression analysis (see Table 3).

Body mass index was highly correlated with AC (*r* = 0.80, *p* < 0.01). However, body mass index was not associated with the incidence of hypotension and significant hypotension. There was a significant univariate correlation between AC and the maximal decrease in MAP after spinal anesthesia (*r* = 0.20, *p* value = 0.04).

## 5. Discussion

In this study, we demonstrated that the incidence of hypotension defined by standard criteria (MAP < 65 mmHg or SBP < 90 mmHg) after spinal anesthesia did not differ between pregnant women with larger and smaller abdominal circumference. However, in the larger AC group, the MAP decreased significantly from baseline. This finding may be attributable to the preemptive treatment of hypotension after spinal anesthesia before the delivery of the newborn. To minimize harm to the fetus caused by uteroplacental hypoperfusion, the anesthesiology team does not normally wait until a pregnant woman develops hypotension [9]. Rather, the general practice is to give an intravenous fluid bolus or administer a vasopressor early whenever there is a trend towards MAP decline or bradycardia. This likely accounts for our failure to identify an increased incidence of hypotension in the larger AC group when we defined hypotension as MAP ≤65 mmHg or SBP <90 mmHg. Nevertheless, the significant decline in MAP from baseline in an individual patient should be concerned because this could lead to hypotension if left untreated. Also, because a systematic review has noted varying definitions of maternal hypotension between trials, we could not restrict defining clinically important hypotension as one specific MAP value [10]. To our knowledge, this is the first research that directly analyzed the impact of the abdominal circumference on the MAP change after spinal anesthesia.

There are many possible mechanisms for the more decline in MAP in pregnant women with larger ACs. First, in a term pregnancy, the uterus is large enough to potentially cause aortocaval compression leading to decreased venous return and cardiac output when lying supine. In pregnant women, the AC reflects the size of the uterus; thus, the larger the AC, the greater the decline in MAP. Second, compression of the inferior vena cava by an enlarged uterus can result in engorgement of the epidural venous plexus, in turn decreasing the cerebrospinal fluid volume and narrowing the intrathecal space [11, 12] potentially resulting in more cephalad spreading of spinal anesthesia and consequently hypotension as a result of the higher degree of sympathectomy [13, 14]. In our study, we did not investigate the effect of the level of spinal anesthesia with larger AC because the aim was to maintain the level of spinal anesthesia at *T*4. We measured the level of anesthesia only after completing the spinal anesthesia and just before the surgical incision. In a previous study, Kuok et al. found a correlation between the AC and sensory block level (right side *ρ* = 0.43, *p*=0.005; left side *ρ* = 0.46, *p*=0.003) [7]. However, those researchers did not find a correlation between the incidence of hypotension, defined as ≥30% decrease of blood pressure from baseline, and AC. The AC from Kuok study was 98.4 ± 6.8 cm, whereas in our study it was slightly larger at 102.79 ± 10.31 cm. We controlled the level of the anesthesia to determine the incidence of hypotension whereas Kuok study aimed at investigating the effects of the level of anesthesia. The Kuok study had a smaller sample size, smaller AC, and different objectives than our study. Zhou et al. found that AC and vertebral column length adjusted for age, weight, and height are the key determinants of the cephalad spread of spinal anesthesia [6]. This principle has also been used to explain the higher sensory block in twin pregnancies than in singleton pregnancies [15]. Third, the size of the abdomen correlates positively with the abdominal pressure [5]. High abdominal pressure has been shown to cause high spinal anesthesia and hypotension [2]. However, we did not measure the abdominal pressure in this study. Thus, we concluded that the decrease in MAP in our study resulted from larger ACs which might be the results of enlarged uteri causing aortocaval compression or increased intra-abdominal pressure. The limitation of this study was the lack of abdominal pressure data which could be used to explain the mechanism of this finding.

The AC can be measured easily and noninvasively and is also nonoperator dependent. Thus, we recommend including this variable to assist anesthesiologists to prepare for hypotensive events after spinal anesthesia in pregnant women. The AC can help to determine which pregnant women should receive early and aggressive hemodynamic treatment. Moreover, we recommend reducing local anesthetic doses on the basis of the AC to reduce the incidence of hypotension [16–18]. Adjusting the dose by body weight is also reportedly useful [19]. Other noninvasive variables such as neonatal size cannot be used to predict the level of anesthesia [20]. One previous study has shown that pregnant women with higher trunk length relative to AC have higher sensory block after spinal anesthesia [21]. We plan to include the trunk length in a future study to further investigate this finding.

## 6. Conclusion

There was no relationship between the incidence of hypotension and abdominal circumference during cesarean section under spinal anesthesia. However, MAP in pregnant women with larger abdominal circumference significantly decreased from baseline after spinal anesthesia.

## Figures and Tables

**Table 1:** Patient characteristics according to the study group.

	Smaller AC group (*n* = 49)	Larger AC group (*n* = 51)	*p* value
^a^Age, years, median (IQR)	29 (27–32)	32 (29–36)	<0.01
Body mass index (kg/m^2^)	24.44 (23.29–27.02)	30.94 (28.32–34.57)	<0.01
^b^ASA physical status			0.67
2	46 (93.9)	49 (96.1)	
3	3 (6.1)	2 (3.9)	
*Hemodynamic variables at baseline*			
Systolic blood pressure (mmHg)	117.81 ± 12.10	126.78 ± 11.79	<0.01
Diastolic blood pressure (mmHg)	69.44 ± 9.42	74.15 ± 12.24	0.03
Mean arterial pressure (mmHg)	85.57 ± 9.44	91.69 ± 10.76	<0.01
Heart rate (beats per minute)	84.16 ± 10.91	89.25 ± 12.18	0.03
*Perioperative data*			
^a^Total operative time (minutes)	75 (65–85)	75 (70–85)	0.60
^a^Blood loss (mL)	400 (300–425)	450 (300–600)	0.04
^a^Total crystalloids used (mL)	1700 (1400–2025)	1600 (1350–1880)	0.39
^a^Urine output (mL)	100 (50–100)	100 (50–200)	0.44
^a^Neonatal weight (g)	3000 (2805–3140)	3430 (3070–3720)	<0.01
Apgar at 1 minute	8 (8–9)	8 (8–9)	0.44
Apgar at 5 minutes	9 (9–10)	9 (9–10)	0.64
^b^Level of anesthesia			0.48
*T*2	1 (2%)	2 (3.9%)	
*T*4	44 (89.8%)	48 (94.1%)	
*T*5	1 (2%)	0	
*T*6	3 (6.1%)	1 (2%)	
^a^Dose of 0.5% bupivacaine heavy (mL)	2.2 (2.2–2.2)	2.2 (2.2–2.4)	0.14

Data are presented as ^a^median (IQR) and ^b^number (%). Data were analyzed using chi-square test and Mann–Whitney *U* test. AC: abdominal circumference; ASA: American Society of Anesthesiologists; IQR: interquartile range.

**Table 2:** Hemodynamic outcomes according to the study group.

		Smaller AC group (*n* = 49)	Larger AC group (*n* = 51)	*p* value
^a^Incidence of hypotension		35 (71.40%)	40 (78.40%)	0.41
^b^Maximal MAP decrease from baseline (mmHg)		28.33 (18.66–33.67)	36.67 (23.33–43.34)	<0.01
^b^Maximal MAP percentage decrease from baseline (percent)		31.41 (22.74–39.22)	38.47 (28.00–44.81)	0.02
^a^Incidence of bradycardia		9 (18.36%)	5 (9.80%)	0.21
^a^Incidence of tachycardia		10 (20.40%)	10 (19.60%)	0.92
^b^Total ephedrine dosage (mg)		18 (6.75–30.00)	21 (15.00–30.00)	0.41

Data are presented as ^a^number (%) and ^b^median (interquartile range). Data were analyzed using chi-square test and Mann–Whitney *U* test. All variables were evaluated 10 minutes after induction of spinal anesthesia. AC: abdominal circumference; MAP: mean arterial pressure.

**Table 3:** Multiple logistic regression analysis of factors associated with significant hypotension after spinal anesthesia in pregnant women.

Independent variables	Crude OR (95% CI)	*p* value	Adjusted OR (95% CI)	*p* value
Larger AC group (versus smaller AC group)	2.73 (1.12–6.65)	0.02	3.67 (1.01–13.28)	0.04
Age	0.98 (0.92–1.05)	0.73	0.98 (0.91–1.05)	0.57
Body mass index	1.04 (0.96–1.14)	0.28	0.98 (0.86–1.11)	0.76
ASA physical status 3 (versus 2)	0.54 (0.05–5.05)	0.54	0.51 (0.04–5.68)	0.58
Baseline heart rate	1.00 (0.96–1.04)	0.87	0.99 (0.95–1.03)	0.84
Intravenous fluid (per mL)	1.00 (0.99–1.00)	0.60	1.00 (0.99–1.00)	0.61
Blood loss (per mL)	0.99 (0.99–1.00)	0.48	0.99 (0.99–1.00)	0.23
Bupivacaine dose (per mL)	1.28 (0.70–2.36)	0.41	1.19 (0.71–2.01)	0.49

Data were analyzed using multiple logistic regression analysis. All variables were evaluated 10 minutes after induction of spinal anesthesia. AC: abdominal circumference; ASA: American Society of Anesthesiologists; OR: odds ratio; CI: confidence interval.

## Data Availability

The data used to support the findings of this study are available on request.
